# Factors Influencing the Crystallization-Onset Time of Metastable ASDs

**DOI:** 10.3390/pharmaceutics14020269

**Published:** 2022-01-23

**Authors:** Friederike Wolbert, Ineke-Katharina Fahrig, Tobias Gottschalk, Christian Luebbert, Markus Thommes, Gabriele Sadowski

**Affiliations:** 1 Drug Delivery innovation Center (DDiC), INVITE GmbH, 51368 Leverkusen, Germany; friederike.wolbert@tu-dortmund.de (F.W.); tobias.gottschalk@tu-dortmund.de (T.G.); 2 Laboratory of Thermodynamics, Department of Biochemical and Chemical Engineering, TU Dortmund University, Emil-Figge-Str. 70, 44227 Dortmund, Germany; ineke.fahrig@tu-dortmund.de (I.-K.F.); christian.luebbert@tu-dortmund.de (C.L.); 3 Laboratory of Solids Process Engineering, Department of Biochemical and Chemical Engineering, TU Dortmund University, Emil-Figge-Str. 68, 44227 Dortmund, Germany; markus.thommes@tu-dortmund.de

**Keywords:** amorphous solid dispersion, hot-melt extrusion, spray drying, stability, water sorption, PC-SAFT, particle size distribution, crystallization kinetics

## Abstract

In formulation development, amorphous solid dispersions (ASD) are considered to improve the bioavailability of poorly water-soluble active pharmaceutical ingredients (APIs). However, the crystallization of APIs often limits long-term stability and thus the shelf life of ASDs. It has already been shown earlier that the long-term stability of ASDs strongly depends on the storage conditions (relative humidity, temperature), the manufacturing methods, and the resulting particle sizes. In this work, ASDs composed of the model APIs Griseofulvin (GRI) or Itraconazole (ITR) and the polymers poly (vinylpyrrolidone-co-vinyl acetate) (PVPVA) or Soluplus^®^ were manufactured via spray drying and hot-melt extrusion. Each API/polymer combination was manufactured using the two manufacturing methods with at least two different API loads and two particle-size distributions. It was a priori known that these ASDs were metastable and would crystallize over time, even in the dry stage. The amount of water absorbed by the ASD from humid air (40 °C/75% relative humidity), the solubility of the API in the ASD at humid conditions, and the resulting glass-transition temperature were predicted using the Perturbed-Chain Statistical Associating Fluid Theory (PC-SAFT) and the Gordon–Taylor approach, respectively. The onset of crystallization was determined via periodic powder X-ray diffraction (PXRD) measurements. It was shown that simple heuristics such as “larger particles always crystallize later than smaller particles” are correct within one manufacturing method but cannot be transferred from one manufacturing method to another. Moreover, amorphous phase separation in the ASDs was shown to also influence their crystallization kinetics. Counterintuitively, phase separation accelerated the crystallization time, which could be explained by the glass-transition temperatures of the evolving phases.

## 1. Introduction

A large number of chemical entities in pharmaceutical research are poorly soluble in water. Their therapeutic effect is limited by their dissolution rate and solubility in the gastrointestinal medium. A promising and widely used approach to formulate these active pharmaceutical ingredients (APIs) is the solution of APIs in amorphous polymers to form a so-called amorphous solid dispersion (ASD) [1,2]. Transforming crystalline APIs into their metastable amorphous state usually improves their dissolution kinetics as well as their solubility in aqueous media. However, APIs are often supersaturated in pharmaceutically relevant polymers and crystallize during storage, limiting the ASD shelf-life [2,3,4,5]. Official approval procedures demand a shelf-life of 6 months at storage conditions of 40 °C/75% relative humidity (RH) or of 12 months at 25 °C/60% RH [6]. ASDs are typically stored in sealed packaging. This means that open-dish storage, as investigated in this work, represents the worst-case scenario.

The prediction and investigation of long-term stability and crystallization kinetics of ASDs are of high interest in current research. In the literature, various research groups deal with the solubility of the API in the polymer and the corresponding thermodynamic stability [7,8,9,10]. Knowing the API solubility in a polymer, it can already be stated whether an API is supersaturated in the ASD or not—i.e., whether or not it crystallizes at infinite time [1,2]. For example, Lehmkemper et al. [8] showed that, for ASDs containing the APIs naproxen and acetaminophen and the polymers poly (vinylpyrrolidone) (PVP) and poly (vinylpyrrolidone-co-vinyl acetate) (PVPVA), the time until crystallization in the ASDs started decreases with increasing temperature, RH, or API content [8]. By considering the phase behavior only, it is not possible to predict at what time the API will crystallize in the ASD (e.g., within 2 h or after 200 years). Therefore, other research groups are particularly interested in crystallization kinetics [11,12,13,14].

Moreover, it is well known but poorly understood that, besides the storage conditions, the manufacturing method (e.g., spray drying/hot-melt extrusion) might significantly impact the long-term stability of ASDs [15,16,17,18,19,20]. Mahmah et al. [15] investigated ASDs of felodipine (FEL) and either PVP K30 or hydroxypropyl methyl cellulose acetate succinate (HPMCAS) with different API loads. The ASDs were prepared via hot-melt extrusion or spray drying. Among other things, they investigated the long-term stability of these ASDs at 40 °C/75% RH. It was observed that the spray-dried FEL/HPMCAS ASD with high FEL loading (w_FEL_ = 0.5) showed crystals after eight weeks of storage, whereas the melt-extruded ASD of the same composition did not. The authors trace this back to the fact that melt-extruded particles are in general more densified than spray-dried particles [15].

Fridgeirsdottir et al. [16] investigated, in total, 60 ASDs of ten different APIs with three different polymers (PVPVA, HPMCAS, and Soluplus^®^) and prepared the ASDs with two different manufacturing methods (spray drying and hot-melt extrusion). They prepared all 60 ASDs with an API load of w_API_ = 10 wt.% and stored the ASDs at 40 °C/75% RH. By determining the crystallization-onset time in those ASDs, they found that spray-dried ASDs containing PVPVA and HPMCAS showed a later crystallization-onset time than the melt-extruded ones; ASDs containing Soluplus^®^ showed the opposite behavior [16]. However, they did not discuss any reasons for this behavior.

Agrawal et al. [17] showed that a spray-dried ASD composed of a not-further-specified API and PVPVA crystallized within one day at 40 °C/75% RH, while the extruded equivalent did not. The same effect was found at 50 °C/51% RH—crystals were observed in the spray-dried ASD after three months of storage, whereas the extruded ASD remained amorphous. The authors explained this finding by the 22-times larger surface area of the spray-dried particles and the resulting higher water adsorption on the particle surfaces [17]. This demonstrates that, in addition to the manufacturing method, as such, the particle size can also be decisive. For example, Kestur et al. [21] investigated the crystallization behavior of FEL powder as a function of particle size. Small particles crystallized faster than larger ones. The authors explained this finding with the larger specific surface area of the small particles [21]. Zhang et al. [22] prepared amorphous simvastatin via two different manufacturing methods (cryo-milling and quench-cooling) in two different particle-size fractions. They found that the cryo-milled simvastatin crystallized faster than the quench-cooled one. Additionally, the small particle fraction (≤10 µm) crystallized faster than the large-particle fraction (150–180 µm) [22].

Consequently, while studying the crystallization kinetics of ASDs, the preparation method and particle size have a significant impact on the results. In this study, we investigated the general validity and transferability of crystallization-kinetics measurement of ASDs to other manufacturing methods and particle sizes. For that purpose, griseofulvin (GRI) and itraconazole (ITR) were selected as model drugs for poorly water-soluble APIs. The solubility of GRI in water is 29.9 µg mL^−1^ (at 37 °C) [23], and the solubility of ITR in water is 2.8 µg mL^−1^ (at 25 °C) [24]. PVPVA and Soluplus^®^ are two of the most commonly used polymers for ASD production. PVPVA is widely used for spray drying; Soluplus^®^ was explicitly developed for hot-melt extrusion.

ASDs with GRI and ITR and the polymers Soluplus^®^ and PVPVA were prepared by two different preparation methods (spray drying and hot-melt extrusion) and with up to three different API loads (10 wt.%, 20 wt.% 40 wt.%). Two different particle sizes were considered for each of the two manufacturing methods. On the one hand, the melt-extruded ASDs were manually ground and then sieved into two particle-size fractions. On the other hand, two different process-parameter sets were used for spray drying to produce ASDs with different particle sizes. The ASD formulations were stored at 40 °C and 75% RH and were repeatedly analyzed for crystallinity by PXRD. This work combines the individual influencing factors of the kind of polymer, production method, particle size, and API content on the crystallization onset time, which were considered separately in previous works. In addition, the results are explained using thermodynamic predictions of solubility, demixing, and the glass-transition temperature at different temperatures and relative humidities.

## 2. Materials and Methods

### 2.1. Materials

**Figure 1:**
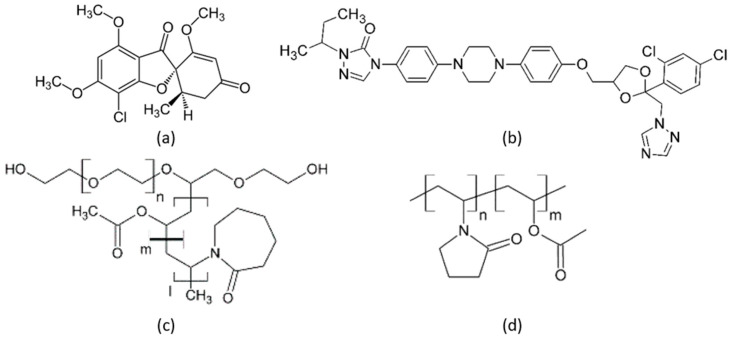
Chemical structures of (**a**) GRI, (**b**) ITR, (**c**) Soluplus^®^, and (**d**) PVPVA.

Griseofulvin (GRI) (Ph. Eur. grade) was purchased from Fagron (Rotterdam, The Netherlands), and itraconazole (ITR) (Ph. Eur. grade) was purchased from Ria International India (Madurai, India). GRI and ITR belong to crystallization tendency class 1 and class 3, respectively [25]. This means that amorphous GRI crystallized in a DSC experiment during cooling before reaching the glass-transition temperature. In contrast, amorphous ITR neither crystallized during cooling below the glass-transition temperature nor during subsequent reheating to the melting point [25]. The polymers PVPVA (Plasdone S-630) with a molecular weight of 40,000 and Soluplus^®^ with a molecular weight of 118,000 g/mol were obtained from Ashland Inc. (Wilmington, DE, USA) and BASF (Ludwigshafen, Germany), respectively. Dichloromethane (purity > 98%) was purchased from VWR (Randor, PA, USA). All substances were used without further purification. Water used for long-term stability tests was filtered and deionized with a Merck Millipore purification system (Darmstadt, Germany). Figure 1 shows the chemical structures of the investigated compounds GRI, ITR, Soluplus^®^_,_ and PVPVA.

### 2.2. Manufacturing of Spray-Dried ASDs

All spray-dried ASDs were manufactured using a mini spray dryer B-290 with inert loop from Büchi (Essen, Germany). The inert gas flow and the API/polymer concentration in the feed were varied to obtain ASDs of two particle-size distributions. Small particles were spray-dried at a nitrogen gas flow of 742 L/h and a solid (API/polymer)/solvent concentration of 10 g/L, whereas large particles were spray-dried at a nitrogen gas flow of 473 L/h and a solid (API/polymer)/solvent concentration of 30 g/L. The volume flow of the feed solution and the relative aspirator power were kept constant at 9 mL/min and 100%, respectively. T_in_ was set to 70 °C for the ASDs containing GRI and set to 60 °C (40 wt.% ITR) or 70 °C (20 wt.% ITR) for ASDs containing ITR. Dichloromethane was used as the solvent. Typically, a residual-solvent content of 2 to 10 wt.% remains in the product [26]. Due to this, all ASDs were dried after spray drying for four days in a vacuum chamber from Binder (Tuttlingen, Germany) at 25 °C/0% RH (vacuum) to perform a secondary drying step.

#### Determination of Particle-Size Distribution

**Figure 2:**
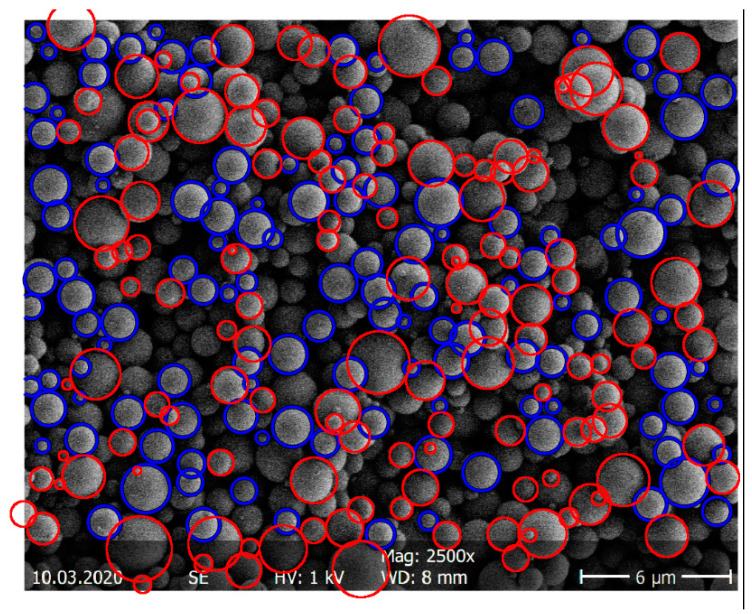
Example determination of the particle-size distribution of an ITR/Soluplus^®^ ASD via MATLAB (blue) and ImageJ (red).

The particle-size distribution of the spray-dried ASDs was determined via SEM imaging (SEM S-4500 by Hitachi, Tokyo, Japan). Statistical image analysis using SEM was performed following ISO 13322-1 (particle size analysis—image analysis methods) [27]. Samples were first fixed as flat as possible on a sample carrier prior to SEM imaging to create a particle monolayer. The SEM image was transformed into a binary image with MATLAB (The MathWorks, Inc., Natick, MA, USA). The MATLAB function “imfindcircles” automatically detected the round particles (size and number). Particles in the upper particle layer undetected by the software were marked manually with the open-source image processing and analysis tool ImageJ [28]. Particles of the layer below were not evaluated due to size distortion. Approximately 1000 particles per sample were examined to determine the particle-size distribution with statistically significant accuracy. This corresponded to an average of six SEM images. The samples were taken from the top, the middle, and the bottom of the sample container in order to represent the entire distribution. Figure 2 shows, for example, the determination of the particles of an ITR/Soluplus^®^ ASD with the automatic evaluation via MATLAB and the manual review via ImageJ.

For statistical evaluation, the particle size distributions were divided into 20 size classes of equal width. The width of a class is the difference of the largest particle diameter to the smallest particle diameter in the class, and the mean particle diameter ${\bar{d}}_{i}$ of a class *i* was the unweighted arithmetic mean. The number distribution$\;Q_{0}\left(d_{i}\right)$ of the spray-dried particles was calculated with Equation (1) [29].

(1) $$Q_{0,i}=\frac{\;\sum_{j=1}^{i}n_{j}}{n_{tot}}$$

Here, $n_{j}$ is the number of particles within the classes 1 to *i*, whereas $n_{tot}$ is the total number of particles measured. To calculate the mean volume of a particle class, the particle shape was assumed to be spherical, which is a perfect assumption for spray-dried particles (compare Figure 2). The mean volume of a particle class $V_{i}$ was then calculated from the mean particle diameter ${\bar{d}}_{i}$ of each class and the number $n_{i}$ of particles in the class *i* following Equation (2) [29].

(2) $$V_{i}=n_{i}\cdot \;\frac{\pi }{6}\cdot {\bar{d}}_{i}^{3}$$

The relative class volume $\Delta Q_{3,i}$ and the volume distribution of the spray-dried particles $Q_{3,i}$ were calculated analogously to the number distribution according to Equations (3) and (4) [29].

(3) $$\Delta Q_{3,i}=\frac{V_{i}}{V_{tot}}$$

(4) $$Q_{3,i}=\overset{i}{\underset{j=1}{\sum }}\Delta Q_{3,j}$$

The density distribution *q*_3,*i*_ was calculated by dividing the relative class volume $\Delta Q_{3,i}$ by the class width. The median value or 50% quantile *d*_50,3_ indicates the particle diameter, below or above which half of the measured particles were found [29].

#### 2.3. Manufacturing of Melt-Extruded ASDs

The melt-extruded ASDs were manufactured with a Micro Compounder Extruder by Xplore Instruments BV (Geleen, The Netherlands). In contrast to industrial extruders, the material can be recirculated in the extruder via a bypass and is only released when the outlet valve is opened. For each extrusion, 3 g of an API-polymer mixture was filled into the extruder via the hopper (to prevent the hopper from heating up, it was cooled by compressed air). The API–polymer mixture was heated to the desired temperature via six individual heating elements in the extruder. The circulation time was set to three minutes. The speed of the screws was set to 50 rpm. The process temperature was set to a temperature above both glass transition and solubility temperature of the API in the polymer and below the decomposition temperature of the polymer. ASDs containing Soluplus^®^ were extruded at 180 °C, and ASDs containing PVPVA were extruded at 190 °C. Subsequently, the extrudate strands were manually ground in a mortar and sieved into two sieve fractions. The ground particles of the small sieve fraction had a diameter between 125 µm and 200 µm, and the particles of the larger sieve fraction had a diameter between 200 µm and 355 µm. After that, the ASDs were dried in a vacuum chamber for four days to ensure comparability among all ASDs.

#### 2.4. Long-Term Stability Tests

All ASD samples (melt-extruded and spray-dried) were stored at 40 °C/75% RH (open dish) in a climate chamber WKL 34 by Weiss (Reiskirchen-Lindenstruth, Germany) to investigate their long-term stability. The PXRD device MiniFlex 600 by Rigaku (Tokyo, Japan) was used to detect the onset of API crystallization in the ASDs. The measurement was performed with the powders purred onto a silicon sample carrier covering a double diffraction angle of 5° < 2Θ < 30° at a tube voltage of 40 kV and an electric current of 15 mA. The measuring speed was set to 5° min^−1^ with a step size of 0.02°. The crystallization onset point was determined by integration over the baseline. For example, pure GRI showed the highest peak intensity at a double diffraction angle of 16°, and the first peak in the amorphous halo appeared at 16°. For this reason, integration was performed after subtracting the baseline between 15° and 18°. Amorphous ASDs exhibited an area of 0 to 0.01. The time at which the integral was greater than 0.01 was assumed to be crystallization-onset time.

It must be noted that the PXRD has a detection limit of up to 5% crystals in the sample [30], depending on the crystallite size. GRI ASDs were repeatedly measured every two to three hours during the first 6 h. The other ASDs were initially examined daily and afterward examined in larger intervals.

## 3. Results and Discussion

### 3.1. Particle Sizes

**Figure 3:**
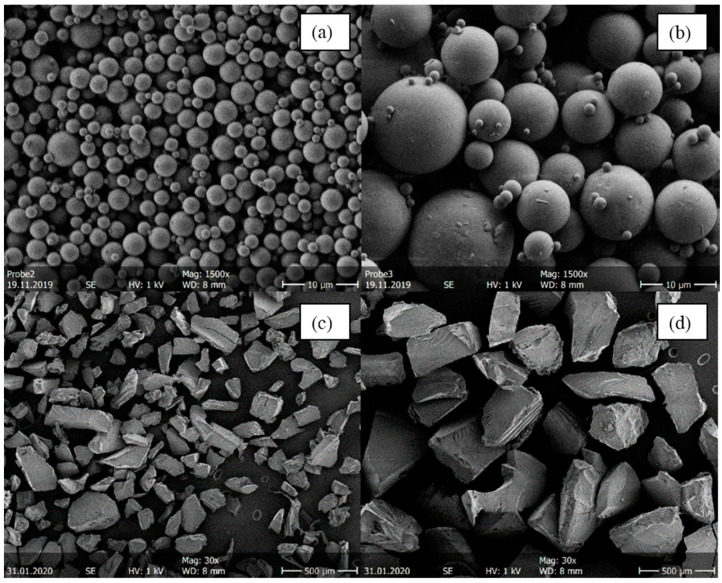
SEM images of four ITR/Soluplus^®^ ASDs with 20 wt.% ITR. (**a**) Small spray-dried particles (d_3,50_ = 2.43 µm), (**b**) large spray-dried particles (*d*_3,50_ = 15.28 µm), (**c**) small melt-extruded particles ( $\bar{d}=162.5\;\mathrm{µm}$), and (**d**) large hot meld extruded particles ($\bar{d}=162.5\;\mathrm{µm}$). Spray-dried samples were imaged with 1500× magnification, melt-extruded samples with 30× magnification.

It was not possible to generate the same particle sizes for melt-extruded ASDs and spray-dried ASDs. The melt-extruded particles could not be ground to such small particles as obtained from the spray dryer without risking crystallization or degradation. Figure 3 shows as an example SEM images of four ITR/SOL samples comparing the particle sizes for spray-dried and melt-extruded ASD particles.

The spray-drying process yields spherical particles [1,2] (Figure 3a,b). By adjusting the spray-drying process parameters (API/polymer concentration in the feed and the gas flow), it was possible to produce different particle-size distributions. Both the small and the large spray-dried particles tend to agglomerate. Agglomerations and thus electrostatic charges can lead to deflection of the electron beam, which makes the measurement by SEM difficult. Furthermore, determining the particle diameters in MATLAB is more difficult for agglomerated systems. The overall mean diameter obtained for spray-dried particles was 2.45 µm, with a standard deviation of 0.57 µm for small particles and 10.47 µm with a standard deviation of 2.95 µm for large particles. Spray drying at conditions leading to large particles led to wider particle size distributions than process conditions for small particles. When comparing Figure 3a,b with Figure 3c,d, it is noticeable that spray-dried particles were significantly smaller than melt-extruded ASDs. Moreover, melt-extruded particles did not have a spherical morphology but an irregular morphology with distinct break-off edges caused by the mortar. The melt-extruded particles had an overall mean diameter of 162.5 µm in the small sieve fraction (Figure 3c) and 277.5 µm in the large sieve fraction (Figure 3d). Consequently, spray-dried particles had a larger specific surface area than melt-extruded particles.

### 3.2. Crystallization-Onset Time

**Figure 4:**
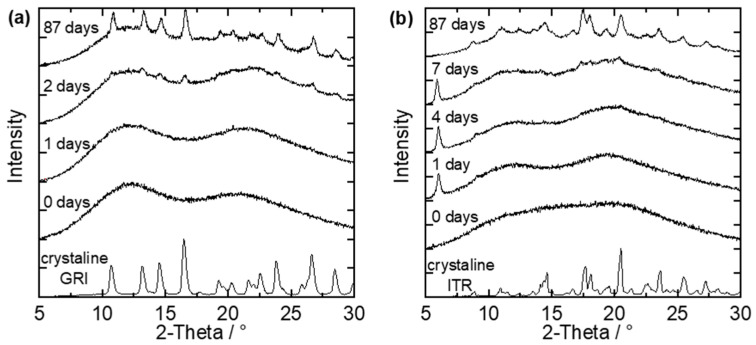
PXRD-diffractograms of (**a**) small particles of a spray-dried GRI/Soluplus^®^ ASD with 20 wt.% GRI and (**b**) small particles of a spray-dried ITR/Soluplus^®^ ASD with 40 wt.% ITR stored at 40 °C/75% RH after different days of storage compared to the diffractograms of pure crystalline GRI and ITR.

The spray-dried and melt-extruded ASDs were stored at 40 °C/75% RH and examined for crystallinity via repeatedly performed PXRD measurements. Figure 4 shows example PXRD diffractograms of two small-particle ASDs, each after a different number of days of storage at 40 °C/75% RH. One example is a spray-dried GRI/Soluplus^®^ ASD with a drug load of 20 wt.% GRI (Figure 4a), and the other is a spray-dried ITR/Soluplus^®^ ASD with a drug load of 40 wt.% ITR (Figure 4b).

On day zero (start day of long-term stability tests), both ASDs were X-ray amorphous (no characteristic peaks in the diffractograms). The first peaks were observable after the second day of storage (Figure 4a). The time between the last amorphous PXRD measurement and the first measurement in which characteristic API-crystal peaks appeared in the PXRD diffractogram was defined as the crystallization-onset time—the exact onset point of crystallization was thus between these two measurements. This means that the crystallization-onset time for the GRI/PVPVA ASD (Figure 4a) was between the first and the second day. The characteristic peaks grow and become more clearly distinguishable from the amorphous halo as time proceeds. After 87 days, the characteristic diffractogram of GRI could be recognized.

ITR can form different polymorphs. The purchased crystalline ITR (lowest line in the diagram) could be assigned to the Form I polymorph [31]. Figure 4b shows two peaks (at 6.05° and 9.05°) already after one day of storage, but these peaks differ from the diffractogram of pure ITR. This API forms liquid crystals, and the two peaks observed after one day belong to the ITR liquid crystal, as shown by Mugheirbi and Tajber [32,33]. In this work, ITR was considered as crystalline only when peaks of the regular ITR crystal were observed in the diffractogram. That means the crystallization-onset time for the example shown in Figure 4b was between the fourth and the seventh day. During further storage, the regular ITR crystal peaks grew and became distinguishable from the amorphous halo. Additionally, the peaks of the liquid crystal shrunk until they were no longer present. For example, only peaks of the regular ITR crystal were observed after 87 days. The recrystallized ITR was again the Form I polymorph of ITR. No polymorph other than the Form I, nor the liquid crystal, was ever detected for ITR within this work [31,32,33].

### 3.3. Long-Term Stability Tests

**Figure 5:**
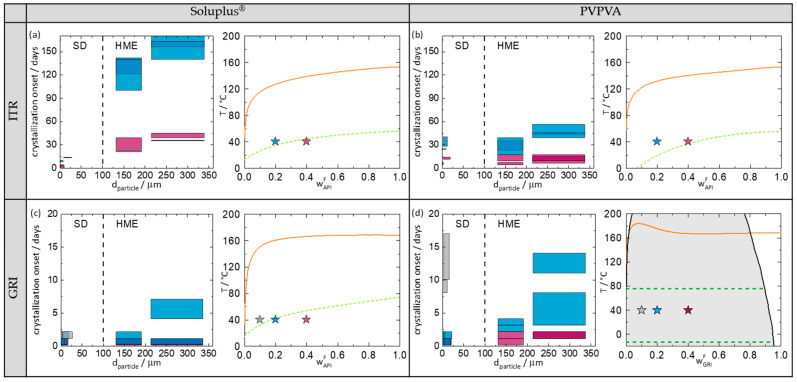
(**a**) ITR/Soluplus^®^, (**b**) ITR/PVPVA, (**c**) GRI/Soluplus^®^, and (**d**) GRI/PVPVA. Grey symbols denotes ASDs with 10 wt.% API, blue symbols denotes ASDs with 20 wt.% API, and purple symbols denotes ASDs with 40 wt.% API. Left site of all subfigures: Crystallization-onset time of ASDs stored at 40 °C/75% RH in days as function of the particle size. The height of the bars marks the period between the last amorphous and first crystalline PXRD measurement (crystallization-onset time). If the crystallization-onset time of two measurements overlap, the bars are superimposed (e.g., darker blue). The width of the bars indicates the particle-diameter range covering 80% of the particles of the ASD. Right site of all subfigures: predicted phase diagrams at 75% RH. The solid lines are PC-SAFT-predicted solubility lines. The dashed lines in (**a**–**c**) are the predicted glass-transition temperatures of the ASDs. The grey area in (**d**) denotes the region of amorphous phase separation calculated with PC-SAFT. The two dashed lines in (**d**) represent the glass-transition temperatures of the two phases (API-rich phase and API-poor phase) evolving in the ASD at 40 °C.

Figure 5 shows the crystallization-onset time plotted against the particle diameter of the investigated ASD. In order to analyze the crystallization results, Figure 5 also shows the phase diagrams of the corresponding ASDs at 75% RH predicted with PC-SAFT (for more information about the theory and the model parameters, see Supplementary Materials) and the location of the investigated ASDs in these phase diagrams.

Figure 5a–d (left) show the crystallization-onset time of the spray-dried and melt-extruded ASDs as function of particle diameter. Spray-dried ASDs and melt-extruded ASDs can be distinguished in the diagrams by the different particle diameters (spray-dried ASDs < 100 µm; melt-extruded ASDs > 100 µm). Moreover, the particle-size distribution of melt-extruded particles is significantly larger than that of spray-dried particles (width of the bars). The width of the particle-size distributions for the melt-extruded particles was determined to cover 80% of the particles excluding outliners at the very low and the very high end of the distribution (all bars have the same width). For the spray-dried particles, the particle size distributions were determined individually for each sample covering 80% of the spray-dried particles (individual width of bars). For each API/polymer combination (a–d), spray-dried ASDs and melt-extruded ASDs, each with two different particle sizes were investigated. The measurements were performed in duplicates.

The right-hand sides of Figure 5 show the phase diagrams indicating the API/polymer combinations from the left-hand diagrams. In Figure 5d, it is noticeable that PC-SAFT predicts an amorphous phase separation for the GRI/PVPVA ASD. This demixing is induced by the absorption of water from the ambient air (there is no demixing in the water-free formulation). Due to amorphous–amorphous phase separation, two phases, a GRI-rich phase and a GRI-poor phase, are expected to evolve [9,34].

Comparing the four phase diagrams (Figure 5), it becomes obvious that the APIs were supersaturated in all investigated ASDs (the symbols are located right of the predicted solubility lines), and the ASDs were thus thermodynamically metastable. As expected, all ASDs crystallized during the study period. The maximum period of being amorphous was 170 days for the melt-extrudate ITR/Soluplus^®^, with 20 wt.% drug load and large particle size (Figure 5a left).

#### 3.3.1. Influence of API Content on Crystallization-Onset Time

To consider the influence of the API content separately from the manufacturing method, the API content is only compared within one manufacturing method. Comparing the four phase diagrams (Figure 5), it can be seen that higher API contents always lead to faster or at least equally fast crystallization (crystallization-onset times: w_API_ = 40 wt.% < w_API_ = 20 wt.% < w_API_ = 10 wt.%). The higher the API content, the higher the supersaturation and thus the faster the crystallization.

#### 3.3.2. Influence of Manufacturing Method on Crystallization-Onset Time

As mentioned above, all ASDs studied in this work were metastable with respect to crystallization. Comparing all ASDs with drug loads of 20 wt.% API, it is noticeable that the melt-extruded ASDs never crystallized faster than the spray-dried ASDs. In three out of four systems, the melt-extruded ASDs had later crystallization-onset times compared to the spray-dried ones. These results are consistent with findings from the literature, that melt-extruded ASDs with PVPVA or HPMCAS crystallized later than spray-dried ones [15,17].

However, in the ITR/PVPVA system (Figure 5b), the ASDs from both manufacturing methods crystallized at almost the same time, although the particle size of the spray-dried ASDs is significantly smaller than that of the melt-extruded ones. Thus, this time, spray drying produced particles that were very stable against crystallization, despite their small particle size. This example clearly shows that there is an influence of the manufacturing method as such, which is not just caused by different particle sizes. Given the fact that the physical behavior of metastable systems always depends on their history (production method), this is not surprising.

#### 3.3.3. Influence of Particle Size on Crystallization-Onset Time

Comparing all melt-extruded ASDs with 20 wt.% API, it is noticeable that ASDs with larger particle sizes had a later crystallization-onset time than the smaller ones. The same holds for spray-dried ASDs with ITR. For GRI ASDs of different particle sizes (Figure 5c,d), the differences in the crystallization-onset times were small since GRI ASDs crystallized much faster than the ITR ASDs due to the higher crystallization tendency of GRI. However, overall, a clear dependence of crystallization-onset time on particle size was observed for all API/polymer combinations. This finding is consistent with similar findings by Zhang et al. [22] and Kestur et al. [21].

However, this clear correlation of crystallization-onset time and particle size is limited to one manufacturing method and cannot be directly transferred to other manufacturing methods. As already mentioned, spray-dried and melt-extruded ITR/PVPVA ASDs crystallized at approximately the same time (Figure 5b), although the particle size of the spray-dried ASDs was significantly smaller than that of the melt-extruded ASDs.

#### 3.3.4. Influence of the Polymer on Crystallization-Onset Time

Comparing the crystallization-onset times of ITR with either Soluplus^®^ or PVPVA, it is noticeable that the ASDs with Soluplus^®^ (Figure 5a) crystallized later than those with PVPVA (Figure 5b). Since the ITR/Soluplus^®^ ASDs were stored closer to their glass-transition temperature compared to the ITR/PVPVA ASDs (e.g., at 20 wt.% API, T-Tg = 5 K for ITR/Soluplus^®^ and T-Tg = 20 K for ITR/PVPVA), this can be explained with the lower molecular mobility in an ASD stored at or below their glass-transition temperature compared to storage above the glass-transition temperature.

PC-SAFT predicts an amorphous phase separation for the GRI/PVPVA ASD (Figure 5d). All three samples were predicted to be located in the demixing area and thus to split up into two liquid phases with the same final compositions (independent of the overall GRI load). Only the ratio of the amorphous phases differs from sample to sample. The drug load of the resulting GRI-rich phase was predicted to be ~98 wt.% GRI. This phase was thus highly supersaturated with respect to GRI crystallization. Nevertheless, all GRI/Soluplus^®^ ASDs crystallized significantly earlier than the GRI-rich phases of the (demixed) GRI/PVPVA ASDs. For example, the melt-extruded GRI/Soluplus^®^ ASD with 20 wt.% GRI (non-demixed) crystallized within 2–7 days, whereas the melt-extruded GRI/PVPVA (demixed) ASD crystallized within 11–14 days.

These findings can again be explained by the glass-transition temperatures. The glass-transition temperature of the GRI-rich phase (upper dashed line in Figure 5d) is approx. 73 °C. This means that at 40 °C, the GRI-rich phases of the three investigated samples were found to be 33 K below their glass-transition temperature. In comparison, the GRI/Soluplus^®^ ASDs with 20 wt.% and 40 wt.% (Figure 5c) were stored much closer to their glass-transition temperature (20 wt.% GRI: T-Tg = −3 K; 40 wt.%: T-Tg = −14 K) and the 10 wt.% GRI/Soluplus^®^ ASD was even stored above its glass-transition temperature. This very well explains the observed crystallization-onset times. Counterintuitively, amorphous demixing was even helpful in stabilizing ASDs against crystallization in this case.

#### 3.3.5. Combined Influence of Manufacturing Method and Polymer on Crystallization-Onset Time

Fridgeirsdottir et al. [16] investigated the combined influence of manufacturing method and polymer on the crystallization onset of ASDs with 10 different APIs (including ITR) and three different polymers. As reported by Fridgeirsdottir et al. [16], spray-dried ASDs with PVPVA showed a later crystallization-onset time than melt-extruded ASDs, whereas spray-dried ASDs with Soluplus^®^ showed an earlier crystallization-onset time than melt-extruded ASDs. For ASDs with ITR (which do not demix), the results of this paper support Fridgeirsdottir et al.’s [16] results. As it can be seen in Figure 5a,b, the spray-dried ITR-PVPVA ASDs showed approximately the same crystallization-onset time as the melt-extruded ASDs (despite having a significantly smaller particle size), whereas the spray-dried ITR-Soluplus^®^ ASDs crystallized much earlier than the melt-extruded ones.

At first glance, the results of this paper for GRI ASDs appear not to correspond to the conclusions from Fridgeirsdottir et al. [16]. As it can be seen in Figure 5d, the spray-dried GRI/PVPVA ASDs with 20 wt.% GRI crystallized up to seven times earlier than the melt-extruded ones. For GRI/Soluplus^®^ with 20 wt.% GRI, the spray-dried ASDs crystallized at the same time or just slightly later (large particles) than the melt-extruded ASDs (Figure 5c). However, as this behavior could be traced back to the amorphous demixing in the ASDs during storage, and Fridgeirsdottir et al. [16] did not investigate those systems, it can be concluded that the results of this work fully agree with those from Fridgeirsdottir et al. [16], as long as the ASDs do not demix.

## 4. Conclusions

In this study, we investigated the general validity and transferability of crystallization-kinetics measurement of ASDs to other manufacturing methods, particle sizes, and polymers. For that purpose, ASDs consisting of either griseofulvin (GRI) or itraconazole (ITR) and either Soluplus^®^ or PVPVA were manufactured via spray drying and hot-melt extrusion with up to three different API weight fractions and two different particle sizes. The differently manufactured ASDs were stored at 40 °C/75% RH and repeatedly examined for crystallinity.

It was shown that higher API contents always lead to faster or at least equally fast crystallization. The higher the API content, the higher the supersaturation, and thus the faster the crystallization. Furthermore, small particles had an earlier crystallization-onset time than bigger ones when using the same manufacturing method. This is consistent with results previously reported in the literature [21,22]. Moreover, considering ASDs of the same composition, none of the melt-extruded ASDs crystallized earlier than the spray-dried ones. Most of them even showed a later crystallization-onset time than the spray-dried ones.

Amorphous phase separation was predicted for GRI/PVPVA ASDs using PC-SAFT. The glass-transition temperature of the evolving GRI-rich phase was predicted to be much higher (73 °C) than the one of the hypothetically homogeneous ASDs (e.g., ~50 °C for 20 wt.% GRI). This led to an increase in long-term stability against crystallization in the demixed ASD compared to the metastable, homogeneous ASD. Compared to the homogeneous GRI/Soluplus^®^ ASDs, the demixed GRI/PVPVA phase also had a much higher glass transition temperature. As a result, the GRI/PVPVA ASDs show a later crystallization-onset time than the GRI/Soluplus^®^ ASDs, regardless of the manufacturing method.

In agreement with earlier studies in the field, it was shown that drug load, glass transition temperature, particle size, and manufacturing method influence the crystallization-onset time of ASDs. A new and so far not considered factor is a possible amorphous phase separation in the ASDs, which was predicted via PC-SAFT. If the glass-transition temperature of the evolving API-rich phase is much higher than that of the hypothetically homogeneous ASD, crystallization might be inhibited at least for certain time periods. Thus, common heuristics are only applicable and transferable for ASDs that do not show amorphous phase separation during preparation and storage.

## Data Availability

The raw data supporting the conclusions of this article will be made available upon request.

## Supplementary Materials

The following supporting information can be downloaded at: https://www.mdpi.com/article/10.3390/pharmaceutics14020269/s1, Chapter 1: Measuring API solubility in the polymer and the glass-transition temperature of the ASD; Chapter 2: Phase diagrams; Table S1: Melting properties of GRI and ITR, the glass-transitions temperatures and amorphous densities of all components; Table S2: PC-SAFT pure-component parameters; Table S3: PC-SAFT binary interaction parameters; Chapter 3: Phase diagrams of water-free ASDs; Figure S1: Phase diagrams of water-free ASDs; Figure S2: Water sorption isotherm of Soluplus at 25 °C; Figure S3: Densities of Soluplus^®^/water mixture at different temperatures; Figure S4: Densities of Soluplus^®^/acetone mixture at different temperatures; Figure S5: Water sorption isotherm of griseofulvin at 25 °C.
