# Supplementary material for: Factors Influencing the Crystallization-Onset Time of Metastable ASDs
Source: Pharmaceutics. 2022 Jan 23;14(2):269. doi: 10.3390/pharmaceutics14020269 (PMC8879851; doi:10.3390/pharmaceutics14020269)
Supplement: Supplementary file 1 [file pharmaceutics-14-00269-s001.zip › pharmaceutics-1558111-supplementary.pdf]

pharmaceutics

Factors Influencing the Crystallization-Onset Time of Metastable ASDs

Friederike Wolbert, Ineke-Katharina Fahrig, Tobias Gottschalk, Christian Luebbert, Markus Thommes and Gabriele Sadowski

1. Measuring API solubility in the polymer and the glass-transition temperature of the ASD

The solubility of GRI and ITR in PVPVA and Soluplus® and the glass-transition temperatures of the corresponding ASDs were measured by modulated differential scanning calorimetry (mDSC) using the Q2000 apparatus from TA Instruments (Eschborn, Germany). The mDSC measurements were conducted with spray-dried ASDs of w_{API} = 20, 40, 60, and 80 wt.%. For each mDSC measurement, 5-10 mg of a sample was filled into a hermetic aluminum pan, sealed with a lid, and placed in the measuring cell, which was purged with 50 mL/min nitrogen. The sample was equilibrated at 25 °C and then heated up to a temperature of 20 K above the melting temperature of the API with a heating rate of 1 K/min, 2 K/min, or 5 K/min and an amplitude of 0.159 K, 0.318 K, or 0.795 K, at a modulation period of 60 s. After keeping the temperature isothermal for 10 min, the sample was cooled down to 20 K below the glass transition temperature of the API with a cooling rate of 20 K/min. The temperature was again kept isothermal for 10 min and then the heating step was repeated (heat-cool-heat procedure).

During the first heating step, the API recrystallized in all samples containing 80 wt.% and 60 wt.% API and sometimes also in those containing 40 wt.% API. In these samples, the melting peak was observed as an endothermic peak in the total heat flow. The offset of the endothermic peak was considered as the solubility temperature of the API in the polymer. The solubility temperature usually linearly depends on the heating rate[1]. The equilibrium solubility temperature was determined by extrapolating the measurement results for 1 K/min, 2 K/min, and 5 K/min to 0 K/min. In the second heating ramp, the glass-transition temperature of the ASD was determined from the half height of the step in the reversing heat flow.

2. Phase diagrams

2.1. Calculation of the API solubility in the polymer at dry and humid conditions

The solubility of an API in the polymer was calculated via a solid-liquid equilibrium. In equilibrium, the chemical potential of the crystalline API (solid phase) is equal to that of the amorphous API dissolved in the polymer (liquid phase). The mole-fraction solubility x_{API}^{L} of the API in the polymer as a function of temperature follows equation (1).[2]

$$x_{API}^{L} = \frac{1}{\gamma_{API}^{L}} exp\left(-\frac{\Delta h_{API}^{SL}}{RT}\left[1-\frac{T}{T_{API}^{SL}}\right]-\frac{\Delta c_{p,API}^{SL}}{R}\left[ln\left(\frac{T_{API}^{SL}}{T}\right)-\frac{T_{API}^{SL}}{T}+1\right]\right) \quad (1)$$

Here Δh_{API}^{SL} is the melting enthalpy, T_{API}^{SL} is the melting temperature, $\Delta c_{p,API}^{SL}$ is the difference in heat capacities of liquid and solid API, and R is the universal gas constant (8.314 J/mol K). Table S1 lists the melting properties of the APIs taken from literature[3]. The activity coefficient of the API in the liquid phase γ_{API}^{L} was calculated using PC-SAFT. It considers intermolecular interactions between the API and surrounding molecules.

The solubility of the API in the polymer is influenced by the absorption of water from humid air. Therefore, additional to the solid-liquid equilibrium, a vapor-liquid equilibrium (chemical potential of water in the air equals the one in the ASD) must be considered at humid conditions. As the vapor pressures of the API and the polymer are negligible

compared to that of water, it was assumed that the vapor-liquid equilibrium only holds for water (equation (2))[4–6].

$$RH = \frac{p_{water}}{p_{0,water}^{LV}} = x_{water}^{L} \cdot \gamma_{water}^{L} \quad (2)$$

RH corresponds to the ratio of the partial pressure of water p_{water} and the vapor pressure of water $p_{0,water}^{LV}$. The latter is a function of temperature. The vapor phase is in equilibrium with the liquid phase and thus RH equals the molar fraction x_{water}^{L} multiplied with the activity coefficient γ_{water}^{L} of water in the liquid. The activity coefficient accounts for the intermolecular interactions among API, polymer, and water and is again calculated using PC-SAFT. Due to the absorption of water, the mass fraction of API and polymer in the ASD change. Nevertheless, the ratio of API to polymer does not change and remains the same as in the dry formulation w_{API}^{F} (equation (3)).

$$w_{API}^{F} = \frac{w_{API}}{w_{API} + w_{polymer}} \quad (3)$$

2.2. Calculation of glass-transition temperatures

The Gordon-Taylor equation was used to calculate the glass-transition temperature of the ASDs T_g^{ASD} (equation (4))[7]. It is a simple mixing rule of the $T_{g,i}$ of the pure components *i* (API, polymer, water) considering their mass fractions w_i and densities ρ_i.

$$T_{g,ASD} = \frac{\sum_i K_i \cdot w_i \cdot T_{g,i}}{\sum_i K_i \cdot w_i} \quad \text{with} \quad K_i = \frac{\rho_{polymer} \cdot T_{g,Polymer}}{\rho_i \cdot T_{g,i}} \quad (4)$$

The Gordon-Taylor parameter K_i was either calculated using the Simha-Boyer rule[8] (equation (4)) or fitted to experimentally data. Table S1 lists the glass-transitions temperatures and densities of all components investigated in this work.

Table S1. Melting properties of GRI and ITR, the glass-transitions temperatures and amorphous densities of all components investigated in this work.

	T^{SL}/ °C	Δh^{SL}/ kJ/mol	Δc_p^{SL}/ J/mol·K	T_g/ °C	ρ/ g/cm^3
GRI	218.02[3]	32.772[3]	93.837[3]	87.75[3]	1.42[9]
ITR	168.75[3]	69.646[3]	177.819[3]	58.16[3]	1.27[10]
PVPVA	-	-	-	108.05[10]	1.19[10]
Soluplus®	-	-	-	68.40[11]	1.14[11]
water				-135.15[12]	1.00

2.3. PC-SAFT

PC-SAFT can be used to predict the activity coefficients needed to calculate the API solubility in the polymer as well as the water-sorption equilibria. The activity coefficients are determined by calculating the residual Helmholtz energy a^{res} of a system according to equation [13].

$$a^{res} = a^{hc} + a^{disp} + a^{assoc} \tag{5}$$

Within PC-SAFT, molecules are considered as a repulsive (hard) chain of m^{seg} spherical segments with diameter σ. a^{hc} describes the contribution of repulsion, whereas a^{disp} is the contribution of attractive forces like van-der-Waals forces. The attraction potential between two molecules is characterized by the dispersion-energy parameter u/k_B (k_B being the Boltzmann constant). Three PC-SAFT pure-component parameters are required (m^{seg} (segment number), σ (segment diameter), u/k_B (dispersion-energy parameter)) to describe non-associating components. The contribution a^{assoc} describes the associative forces like the formation of hydrogen bonds between associating molecules. These interactions are characterized by the parameters association volume κ^{AB} and association energy ε^{AB}/k_B. In the case of associating molecules, five pure-component parameters are required ($m^{seg}, \sigma, u/k_B, \kappa^{AB}, \varepsilon^{AB}/k_B$). The pure-component parameters of all substances considered in this work are listed in Table S2. The Soluplus® pure-component parameters were not available so far and thus fitted to water-sorption isotherms and densities of Soluplus®/water and Soluplus®/acetone mixtures in this work (see supporting information, Figures SI2, SI3, and SI4). All other parameters were taken from literature.

Table S2. PC-SAFT pure-component parameters of compounds investigated in this work.

	M/ g/mol	m^{seg}/M/ mol/g	σ/ Å	u/k_B/ K	ε^{AB}/k_B/ K	κ^{AB}	N^{assoc}
GRI	352.77	0.0402[3]	3.372[3]	221.261[3]	1985.49[3]	0.02[3]	2/2
ITR	705.63	0.0370[3]	2.166[3]	252.346[3]	1204.88[3]	0.02[3]	2/2
PVPVA	47,000	0.0372[14]	2.947[14]	205.271[14]	0[14]	0.02[14]	472/472
Soluplus®	118000	0.0540*	2.809*	225.000*	0*	0.02*	2486/2486
water	18.015	0.0669[15]	σ_{water}#[15]	353.945[15]	2425.67[15]	0.0451[15]	1/1

*this work, #σ_{water}=2.7927+10.11 exp(-0.01775·T/K)-1.417·exp(-0.01146·T/K)[15]

Berthelot-Lorentz mixing rules[16] were applied to calculate the dispersion energy and segment diameter in mixtures. The dispersion energy in a mixture was calculated according to (equation (6)).

$$u_{ij} = (1 - k_{ij})\sqrt{u_i \cdot u_j} \tag{6}$$

The binary interaction parameter k_{ij} corrects the dispersion energy in binary mixtures and was either taken from literature or fitted to experimental data (see supporting

information, Figures S1, S2, S5, and S6). Cross-association among components was described as suggested by Wolbach and Sandler[17]. All binary interaction parameters used in this work are given in Table S3.

Table S3. PC-SAFT binary interaction parameters (k_{ij}) between compounds investigated in this work.

	$k_{ij,int}$
GRI / PVPVA	-0.00407*
GRI/ Soluplus®	-0.00660*
GRI/ water	0.01250*
ITR / PVPVA	-0.05050*
ITR / Soluplus®	-0.02096*
ITR/ water	0.03550*
water / PVPVA	-0.15647#
water / Soluplus®	-0.07800*

*this work #ref. [14]

3. Phase diagrams of water-free ASDs

The measured solubilities and glass-transition temperatures of the investigated API/polymer combinations are shown in Figure S1. In addition, the solubility modeling and the modeling of the glass-transition temperature using the Gordon-Taylor equation are shown. For the ASDs containing ITR (ITR/Soluplus® and ITR/PVPVA), solubility data by Kyeremateng et al.[18] is available and is shown as well.

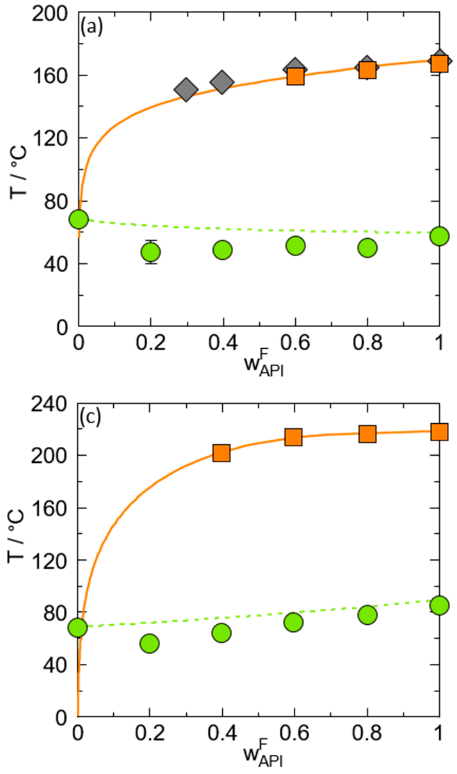

Figure S1. Phase diagrams of water-free ASD formulations (a) ITR/Soluplus®, (b) ITR/PVPVA, (c) GRI/Soluplus® and (d) GRI/PVPVA. Squares and circles show solubility temperatures and the glass-transition temperatures of the ASDs, respectively. The solid lines represent the solubility lines modeled using PC-SAFT. The dashed lines represent the glass-transition temperatures modeled using the Gordon-Taylor equation. The diamonds are experimentally determined solubilities by Kyeremateng et al.[18].

As shown in Figure S1 the experimentally-obtained solubilities can be very precisely described using PC-SAFT and a binary interaction parameter kij between API and polymer fitted to this data. The Gordon-Taylor equation sufficiently well describes the glass-transition temperature. PC-SAFT extrapolations of solubility data to 25°C reveal that the API solubility in the polymer at ambient temperatures is low for all investigated combinations. The solubility of ITR is approx. $1.04\cdot10^{-2}$ wt.% in Soluplus® and $5.48\cdot10^{-3}$ wt.% in PVPVA. The solubility of GRI in Soluplus® is approx. $4.66\cdot10^{-1}$ wt.% and the GRI solubility in PVPVA is $4.90\cdot10^{-1}$wt.%. Moreover, it can be seen from Figure S1 that all API/polymer combinations are supersaturated (right of the solubility line) which was intentionally selected in this work to ensure API crystallization in measurable time.

1. Additional diagrams:

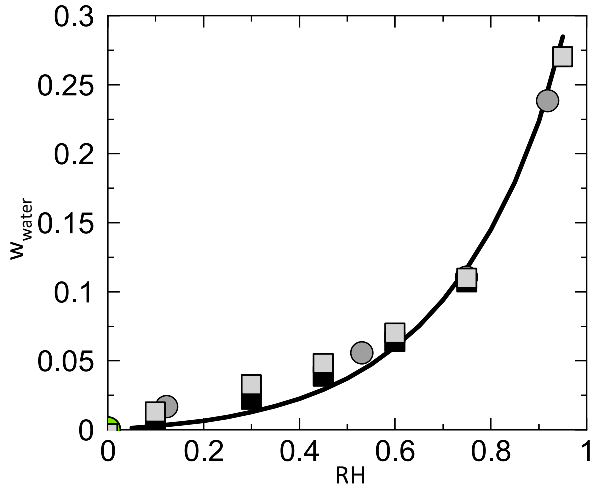

Figure S2. Water sorption isotherm of Soluplus at 25 °C. The Symbols present the experimentally obtained water sorption isotherm (light gray squares: sorption isotherm of this work, black squares: desorption isotherm of this work, gray circles: sorption isotherm from BASF[19] and the line presents the calculated water sorption isotherm with PC-SAFT..

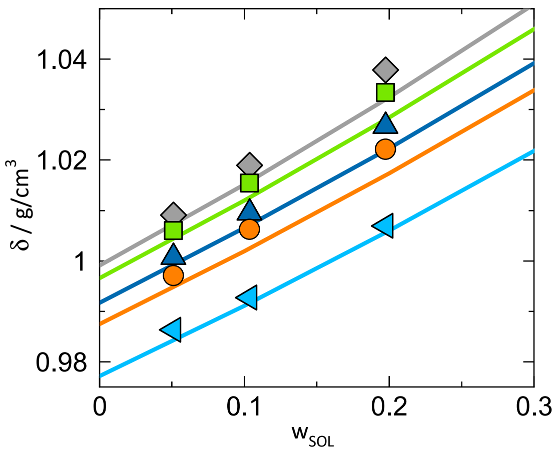

Figure S3. Densities of Soluplus®/water mixture at 10 °C (light blue), 25 °C (orange), 40 °C (dark blue), 50 °C (green) and 70 °C (gray). Symbols are experimentally obtained densities, the lines are calculated with PC-SAFT.

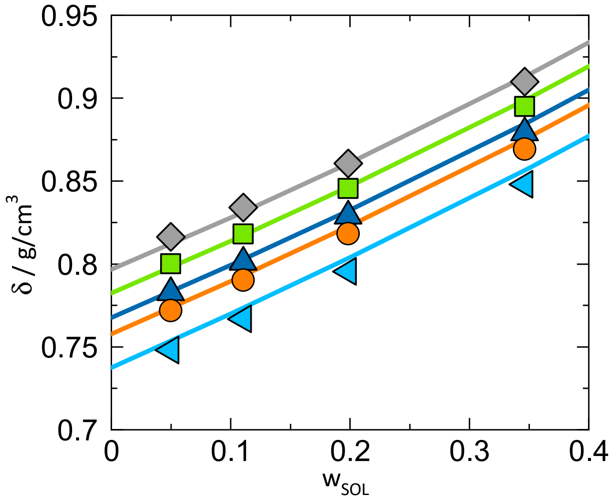

Figure S4. Densities of Soluplus®/acetone mixture at 10 °C (light blue), 25 °C (orange), 40 °C (dark blue), 50 °C (green) and 70 °C (gray). Symbols are experimentally obtained densities, the lines are calculated with PC-SAFT.

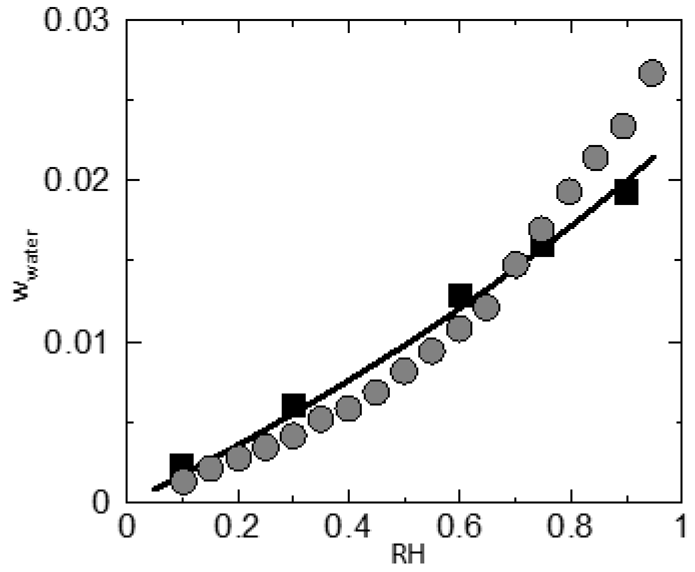

Figure S5. Water sorption isotherm of griseofulvin at 25 °C. The Symbols present the experimentally obtained water sorption isotherm (gray circles: Ozaki et at.[20], black squares: this work) and the line presents the calculated water sorption isotherm with PC-SAFT.

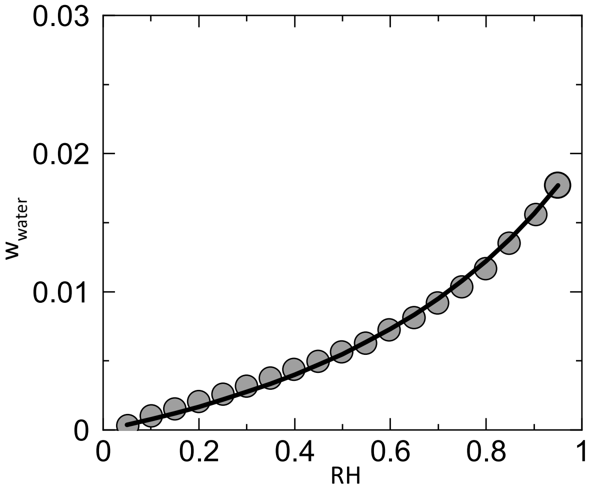

Figure S6. Water sorption isotherm of itraconazole at 25 °C. The Symbols present the experimentally obtained water sorption isotherm from Ozaki et al. [20] and the line presents the calculated water sorption isotherm with PC-SAFT.

References

1. Prudic, A.; Ji, Y.; Sadowski, G. Thermodynamic phase behavior of API/polymer solid dispersions. *Mol. Pharm.* **2014**, *11*, 2294–2304, doi:10.1021/mp400729x.
2. Prausnitz, J.M.; Azevedo, E.G.d.; Lichtenthaler, R.N. *Molecular thermodynamics of fluid-phase equilibria*, 3rd ed.; Prentice Hall PTR: Upper Saddle River, N.J, 1999, ISBN 978-0139777455.
3. Paus, R.; Ji, Y.; Vahle, L.; Sadowski, G. Predicting the Solubility Advantage of Amorphous Pharmaceuticals: A Novel Thermodynamic Approach. *Mol. Pharm.* **2015**, *12*, 2823–2833, doi:10.1021/mp500824d.
4. Prudic, A.; Ji, Y.; Luebbert, C.; Sadowski, G. Influence of humidity on the phase behavior of API/polymer formulations. *Eur. J. Pharm. Biopharm.* **2015**, *94*, 352–362, doi:10.1016/j.ejpb.2015.06.009.

5. Luebbert, C.; Sadowski, G. Moisture-induced phase separation and recrystallization in amorphous solid dispersions. *Int. J. Pharm.* **2017**, *532*, 635–646, doi:10.1016/j.ijpharm.2017.08.121.
6. Luebbert, C.; Wessner, M.; Sadowski, G. Mutual Impact of Phase Separation/Crystallization and Water Sorption in Amorphous Solid Dispersions. *Mol. Pharm.* **2018**, *15*, 669–678, doi:10.1021/acs.molpharmaceut.7b01076.
7. Gordon, M.; Taylor, J.S. Ideal copolymers and the second-order transitions of synthetic rubbers. i. non-crystalline copolymers. *J. Appl. Chem.* **1952**, *2*, 493–500, doi:10.1002/jctb.5010020901.
8. Simha, R.; Boyer, R.F. On a general relation involving the glass temperature and coefficients of expansion of polymers. *J. Chem. Phys.* **1962**, *37*, 1003–1007.
9. Zhou, D.; Zhang, G.G.Z.; Law, D.; Grant, D.J.W.; Schmitt, E.A. Thermodynamics, molecular mobility and crystallization kinetics of amorphous griseofulvin. *Mol. Pharm.* **2008**, *5*, 927–936, doi:10.1021/mp800169g.
10. Six, K.; Verreck, G.; Peeters, J.; Brewster, M.; van den Mooter, G. Increased physical stability and improved dissolution properties of itraconazole, a class II drug, by solid dispersions that combine fast- and slow-dissolving polymers. *J. Pharm. Sci.* **2004**, *93*, 124–131, doi:10.1002/jps.10522.
11. Rask, M.B.; Knopp, M.M.; Olesen, N.E.; Holm, R.; Rades, T. Comparison of two DSC-based methods to predict drug-polymer solubility. *Int. J. Pharm.* **2018**, *540*, 98–105, doi:10.1016/j.ijpharm.2018.02.002.
12. Hallbrucker, A.; Mayer, E.; Johari, G.P. The heat capacity and glass transition of hyperquenched glassy water. *Philos. Mag. B* **1989**, *60*, 179–187, doi:10.1080/13642818908211189.
13. Gross, J.; Sadowski, G. Perturbed-Chain SAFT: An Equation of State Based on a Perturbation Theory for Chain Molecules. *Ind. Eng. Chem. Res.* **2001**, *40*, 1244–1260, doi:10.1021/ie0003887.
14. Lehmkemper, K.; Kyeremateng, S.O.; Heinzerling, O.; Degenhardt, M.; Sadowski, G. Long-Term Physical Stability of PVP- and PVPVA-Amorphous Solid Dispersions. *Mol. Pharm.* **2017**, *14*, 157–171, doi:10.1021/acs.molpharmaceut.6b00763.
15. Cameretti, L.F.; Sadowski, G. Modeling of aqueous amino acid and polypeptide solutions with PC-SAFT. *Chem. Eng. Process.* **2008**, *47*, 1018–1025, doi:10.1016/j.cep.2007.02.034.
16. Calvin, D.W.; Reed, T.M. Mixture Rules for the Mie (n, 6) Intermolecular Pair Potential and the Dymond–Alder Pair Potential. *J. Chem. Phys.* **1971**, *54*, 3733–3738, doi:10.1063/1.1675422.
17. Wolbach, J.P.; Sandler, S.I. Using Molecular Orbital Calculations To Describe the Phase Behavior of Cross-associating Mixtures. *Ind. Eng. Chem. Res.* **1998**, *37*, 2917–2928, doi:10.1021/ie970781l.
18. Kyeremateng, S.O.; Pudlas, M.; Woehrle, G.H. A Fast and Reliable Empirical Approach for Estimating Solubility of Crystalline Drugs in Polymers for Hot Melt Extrusion Formulations. *J. Pharm. Sci.* **2014**, *103*, 2847–2858, doi:10.1002/jps.23941.
19. BASF Pharma Ingredients & Service. *Soluplus*: *Technical Information*, 2009.
20. Ozaki, S.; Kushida, I.; Yamashita, T.; Hasebe, T.; Shirai, O.; Kano, K. Evaluation of drug supersaturation by thermodynamic and kinetic approaches for the prediction of oral absorbability in amorphous pharmaceuticals. *J. Pharm. Sci.* **2012**, *101*, 4220–4230, doi:10.1002/jps.23306.
